# Mental Health Appointments in the Era of COVID-19: Experiences of Patients and Providers

**DOI:** 10.31486/toj.21.0039

**Published:** 2021

**Authors:** Natalie Hunsinger, Rebecca Hammarlund, Kathleen Crapanzano

**Affiliations:** ^1^ Department of Psychiatry, LSU-OLOL Psychiatry Residency Program, Louisiana State University Health Sciences Center–Baton Rouge, Baton Rouge, LA; ^2^ Department of Academic Affairs, LSU-OLOL Psychiatry Residency Program, Our Lady of the Lake Regional Medical Center, Baton Rouge, LA

**Keywords:** *COVID-19*, *mental health appointments*, *telemedicine*

## Abstract

**Background:** The coronavirus disease 2019 (COVID-19) pandemic brought an unprecedented shift in health care toward telepsychiatry. This worldwide phenomenon was necessary to meet community health needs while prioritizing patient and provider safety. This study explored the impact of changes in delivery of mental health care services during the pandemic on patient and provider satisfaction with care.

**Methods:** Providers and patients at an academic outpatient psychiatric clinic completed an electronic, cross-sectional, anonymous survey. Items probed perceived convenience and comfort with in-person and telehealth visits, COVID-19–related depression and anxiety, and visit modality preferences.

**Results:** The response rate was 80.0% for providers and 21.0% for patients. Providers found telehealth more convenient than in-person visits during the pandemic, *t*(11)=1.66, *P*=0.024. Patients reported no differences in convenience (*P*=0.497) or comfort (*P*=0.535) ratings. As the pandemic continues, 83.3% of providers and 50.0% of patients indicated they would prefer telehealth visits. Only 16.7% of providers and 25.0% of patients would prefer telehealth visits continue after the pandemic. Preferred appointment type during the pandemic was not significantly correlated with pandemic-related anxiety or depression.

**Conclusion:** Providers showed a strong preference for telehealth visits during the pandemic, whereas patients felt equal convenience and comfort with care in both telehealth and in-person visits. Fewer participants preferred to use the telehealth modality after the pandemic ends. Higher COVID-19–related depression or anxiety did not impact preference toward visit type. These results suggest that telepsychiatry is an acceptable, although not always preferred, modality for psychiatric care during the global pandemic.

## INTRODUCTION

The coronavirus disease 2019 (COVID-19) pandemic caused an unprecedented shift in health care toward telemedicine, including telepsychiatry. This shift was necessary to meet community health needs while prioritizing the safety of patients and providers.^1-4^ Previous research comparing telepsychiatry services to in-person care found telepsychiatry services comparable to in-person services in many facets, including patient satisfaction, provider assessment, and treatment outcomes.^5-8^ These findings suggest that even with the shift to telepsychiatry services during the pandemic, adequate care can be provided to patients. However, other influences may impact perceptions and efficacy of care during the pandemic. For example, health care clinics modified their in-person services during the pandemic with substantial changes, including mandatory masking, temperature checks and other screening procedures, and social distancing requirements. Furthermore, the pandemic has increased community anxiety about the safety of being in public spaces and health care areas*.* The impact of these changes on the delivery of care in clinics remains unclear.

The primary purpose of the current study was to investigate how changes in the delivery of mental health care services (ie, telehealth visits and modified in-person visits), necessitated by the COVID-19 pandemic, have impacted provider and patient satisfaction in terms of convenience of visits and comfort with the care provided. A secondary aim of this study was to assess the impact of the pandemic on feelings of anxiety and depression in both providers and patients and to investigate associations between these feelings and preferences for mental health visit modalities during and after the pandemic.

We had several hypotheses for this study. Our first hypothesis was that providers and patients would rate telehealth visits as more convenient than in-person visits during the pandemic. Our second hypothesis was that providers and patients would find telehealth visits during the pandemic to be as comfortable as in-person visits that occurred prior to the pandemic. Regarding visit type preferences, our third hypothesis was that most providers and patients would prefer to continue telehealth visits for the duration of the pandemic, whereas preferences for appointment type after the pandemic would be more mixed. Our final hypothesis was that individuals experiencing higher levels of depression or anxiety during the pandemic would tend to prefer telehealth visits to in-person visits during the pandemic.

## METHODS

This study was approved by the institutional review board of Louisiana State University Health Sciences Center. Written informed consent was not obtained to preserve anonymity of the data; however, before completing the survey, all participants read an information page that contained similar information to an informed consent, including the purpose of the study, the risks and benefits of participating, and researcher contact information. Participants were notified that continuing with the survey and responding to items indicated their consent to participate.

The study consisted of a cross-sectional, anonymous survey conducted online via REDCap (Vanderbilt University).^9^ Invited participants were established patients and mental health care providers at an academic outpatient psychiatry clinic. The following inclusion criteria were used in patient recruitment: adults ≥18 years; established patients of the outpatient clinic prior to the pandemic (ie, had an in-person appointment prior to March 23, 2020, the date the study clinic initiated widescale changes to clinic procedures in response to the pandemic); patients who recently attended at least 1 pandemic-altered visit, either via telehealth or under new in-person procedures; and patients who had an activated MyChart (Epic Systems Corp) account. MyChart, a patient messaging portal that is compliant with the US Health Insurance Portability and Accountability Act, was used for communication for study recruitment and distribution of study and survey information. All patients and providers participated in in-person visits prior to the start of the pandemic. Telepsychiatry services in the study site clinic were minimal prior to onset of the pandemic, with only 2 providers providing some telepsychiatry services to established patients.

All outpatient providers of adult psychiatric services at the study site were invited to participate in the study on a voluntary basis. Provider participants included psychiatrists, psychiatric residents, and an advanced practice psychiatric mental health nurse practitioner.

Following identification of eligible patients in the electronic medical record system (Epic Systems Corp), investigators sent an electronic invitation to participate containing the link to the survey via MyChart. Invitations were sent on a rolling basis at weekly intervals from September 15, 2020 until December 11, 2020. All potential provider participants were sent an invitation to the study with the survey link via their secure work email address. Study data were collected and managed using REDCap.^9^

Two versions of the survey were created: 1 for providers and 1 for patients. Branching logic was used to direct participants to the appropriate questions, depending on the participant type (patient or provider) and visit type for patients (telehealth or in-person). For both groups, the only demographic items collected were age (in terms of ranges) and sex. Both items included a “prefer not to answer” option. Patient participants were asked to rate the convenience of their most recent visit on a 5-point scale from “very convenient” to “very inconvenient.” Participants who indicated their visit was “somewhat inconvenient” or “very inconvenient” were further asked to indicate what specific factor(s) led to their rating by selecting from provided options and/or specifying their own unlisted factors. In addition to convenience, participants were asked to rank their comfort with their interaction with their provider during the most recent appointment, as compared with in-person visits conducted before the pandemic. This item was also ranked on a 5-point scale from “much easier or more comfortable” to “much more difficult or uncomfortable.” Participants were then asked to indicate what type of appointments they prefer during the pandemic and what type they would prefer after the pandemic ends. Finally, anxiety and depression were probed with 2 items that asked the participant to rank their symptoms on a 5-point scale from “much less anxiety/depression than normal for me” to “much more anxiety/depression than normal for me.” Participants were also given a “prefer not to answer” option for these items.

The provider survey was virtually identical to the patient survey, with 2 exceptions. First, all providers were asked about both types of appointments (telehealth and in-person), because as providers, they had conducted both. Second, providers were asked to rate their global impressions about their patients’ levels of anxiety and depression during the pandemic in addition to their own personal levels of such symptoms. This item was rated on the same 5-point scale.

Descriptive statistics are used to summarize how participants responded to survey items. With 3 being the neutral point of the scale, ratings >3 indicate more negative responses (ie, toward inconvenience, discomfort, increased anxiety, or depression), while ratings <3 indicate more positive responses. Independent samples *t* tests were used to compare patients’ and providers’ mean responses to analogous survey items (ie, appointment convenience, comfort with the interaction); paired samples *t* tests were used to compare providers’ responses across appointment types; and one sample *t* tests were used to compare comfort levels with a theoretical neutral rating of 3. Relationships among variables were also explored with correlations and chi-square test analyses as appropriate.

## RESULTS

During the 3-month recruitment period, 15 providers and 334 patients met inclusion criteria and were invited to participate. Overall, 13 providers and 83 patients completed at least part of the survey. However, review of these records revealed that 1 provider and 12 patients did not complete enough items to be included in the data analyses, so these participants were dropped. The final response rate was 80% (12 of 15) for providers and 21% (71 of 334) for patients. Within the patient group, 3 patients did not fully answer visit preference questions (therefore, n=68 for visit preferences results noted below); however, they answered the remaining items, so we retained their surveys.

Demographics and appointment types of the sample are summarized in Table 1. Among patients, 31 attended telehealth visits, while 40 attended in-person visits during the pandemic. All 12 provider respondents conducted both in-person and telehealth visits during the pandemic.

**Table 1.** Demographics and Appointment Types for Patients and Providers

Variable	Patients, n=71	Providers, n=12
Age range, years		
18-25	12 (16.9)	0
26-35	10 (14.1)	7 (58.3)
36-45	12 (16.9)	1 (8.3)
46-55	9 (12.7)	0
56-65	14 (19.7)	3 (25.0)
66+	14 (19.7)	0
Prefer not to answer	0	1 (8.3)
Sex		
Male	13 (18.3)	4 (33.3)
Female	55 (77.5)	7 (58.3)
Nonbinary	3 (4.2)	1 (8.3)
Prefer not to answer	0	0
Appointment type		
Telehealth	31 (43.7)	12 (100)
In-person	40 (56.3)	12 (100)

Note: Data are presented as n (%).

Table 2 shows response frequencies for the telehealth and in-person visit convenience ratings, as well as the means, SDs, and statistical test results. A majority of patients found both visit types “very convenient.” Ratings did not differ significantly between the telehealth and in-person visit patients (comparison *a vs b, P*=0.497). In-person visits during the pandemic were rated as more convenient by patients than by providers, (comparison *b vs d, P*<0.001), whereas no statistical difference was found between group ratings of the convenience of telehealth appointments (comparison *a vs c, P*=0.298). Within-subjects comparison for providers showed that telehealth visits were rated as significantly more convenient than in-person visits (comparison *c vs d, P*=0.024).

**Table 2.** Convenience Rating Frequencies, Means ± SD, and Group Comparisons

Convenience Ratings and Comparisons by Group	n (%)		Mean ± SD
**Patients**			
Telehealth visits (n=40)^a^			1.68 ± 1.23
Very convenient	28 (70.0)		
Somewhat convenient	5 (12.5)		
Neither	1 (2.5)		
Somewhat inconvenient	4 (10.0)		
Very inconvenient	2 (5.0)		
In-person visits (n=31)^b^			1.48 ± 1.09
Very convenient	25 (80.6)		
Somewhat convenient	1 (3.2)		
Neither	2 (6.5)		
Somewhat inconvenient	2 (6.5)		
Very inconvenient	1 (3.2)		
**Providers**			
Telehealth visits (n=12)^c^			2.08 ± 0.99
Very convenient	3 (25.0)		
Somewhat convenient	7 (58.3)		
Neither	0		
Somewhat inconvenient	2 (16.7)		
Very inconvenient	0		
In-person visits (n=12)^d^			3.33 ± 0.89
Very convenient	1 (8.3)		
Somewhat convenient	0		
Neither	5 (41.7)		
Somewhat inconvenient	6 (50.0)		
Very inconvenient	0		
**Comparisons**	***t***	***P* value**	**95% CI**
^a^ ^vs^ ^b^*Patient telehealth vs patient in-person visits	–0.68	0.497	–0.75 to 0.37
*^a^ ^vs^ ^c^*Patient telehealth vs provider telehealth visits	–1.05	0.298	–1.19 to 0.37
*^b^ ^vs^ ^d^*Patient in-person vs provider in-person visits	–5.23	<0.001	–2.56 to –1.14
^c^ ^vs^ ^d^*Provider telehealth vs provider in-person visits	1.66	0.024	0.20 to 2.30

*Within-subjects comparison.

Half of providers (50%, n=6) rated in-person appointments as “somewhat inconvenient” and cited mask wearing, no-shows, finding childcare, screening procedures, and finding space to work as factors that made these visits inconvenient. Only 2 (16.7%) providers felt telehealth appointments were “somewhat inconvenient” and endorsed equipment, internet, and software problems as inconveniences. Among patients, only 3 (9.7%) rated in-person appointments as “somewhat” or “very” inconvenient, with cited convenience factors including scheduling, transportation, and mask wearing. For telehealth appointments, 6 (15.0%) patients rated visits as “somewhat” or “very” inconvenient, citing equipment issues or “other” responses including anxiety, not knowing if the patient should initiate the call, and dislike of video calls.

Table 3 shows response frequencies for telehealth and in-person appointment comfort ratings as well as the means, SDs, and statistical test results. On average, patients rated interactions with their providers during both telehealth and in-person visits as “somewhat easier or more comfortable” than during their prepandemic in-person appointments. These ratings did not differ between the 2 groups (comparison *a vs b, P*=0.535). In contrast, providers differed significantly in their ratings for the 2 types of appointments (*c vs d, P*=0.004).

**Table 3.** Comfort Rating Frequencies, Means ± SD, and Group Comparisons

Comfort Ratings and Comparisons by Group	n (%)		Mean ± SD	
Patients*				
Telehealth visits (n=38)^a^			2.16 ± 1.13	
Much easier or more comfortable	12 (31.6)			
Somewhat easier or more comfortable	16 (42.1)			
Just as difficult or uncomfortable	3 (7.9)			
Somewhat more difficult or uncomfortable	6 (15.8)			
Much more difficult or uncomfortable	1 (2.6)			
In-person visits (n=30)^b^			2.00 ± 0.91	
Much easier or more comfortable	9 (30.0)			
Somewhat easier or more comfortable	15 (50.0)			
Just as difficult or uncomfortable	3 (10.0)			
Somewhat more difficult or uncomfortable	3 (10.0)			
Much more difficult or uncomfortable	0			
**Providers**				
Telehealth visits (n=12)^c^			2.58 ± 0.79	
Much easier or more comfortable	0			
Somewhat easier or more comfortable	0			
Just as difficult or uncomfortable	3 (25.0)			
Somewhat more difficult or uncomfortable	9 (75.0)			
Much more difficult or uncomfortable	0			
In-person visits (n=12)^d^			3.75 ± 0.45	
Much easier or more comfortable	0			
Somewhat easier or more comfortable	7 (58.3)			
Just as difficult or uncomfortable	3 (25.0)			
Somewhat more difficult or uncomfortable	2 (16.7)			
Much more difficult or uncomfortable	0			
**Comparisons**	***t***	***P value***	**95% CI**	
^a^ ^vs^ ^b^**Patient telehealth vs patient in-person visits	–0.62	0.535	–0.66 to 0.35	
*^a^ ^vs^ ^c^*Patient telehealth vs provider telehealth visits	–1.21	0.232	–1.13 to 0.28	
*^b^ ^vs^ ^d^*Patient in-person vs provider in-person visits	–6.33	<0.001	–2.31 to –1.19	
^c^ ^vs^ ^d^**Provider telehealth vs provider in-person visits	3.63	0.004	0.46 to 1.88	

*Missing data points: 2 responses for telehealth patients and 1 response for in-person patients.

**Within-subjects comparison.

Patient- and provider-reported preferences regarding visit type during and after the pandemic are outlined in Table 4. The patients’ recent visit type was related to their preferences during (χ^2^[2]=25.74, *P*<0.001) and after (χ^2^[2]=13.44, *P*=0.001) the pandemic. During the pandemic, recent telehealth patients preferred to continue with telehealth, whereas recent in-person patients were split between in-person and no preference. After the pandemic, recent telehealth patients were more split between in-person and telehealth visits, while recent in-person patients’ preferences were the same.

**Table 4.** Visit Type Preference Frequencies During and After the Pandemic

		Patients^a^		
Time Period	Preference	Seen in Person, n=30	Seen via Telehealth, n=38	Providers, n=12
During the pandemic	Prefer in-person	13 (43.3)	2 (5.3)	1 (8.3)
	Prefer telehealth	5 (16.7)	29 (76.3)	10 (83.3)
	No preference	12 (40.0)	7 (18.4)	1 (8.3)
After the pandemic	Prefer in-person	17 (56.7)	13 (34.2)	4 (33.3)
	Prefer telehealth	1 (3.3)	16 (42.1)	2 (16.7)
	No preference	12 (40.0)	9 (23.7)	6 (50.0)

^a^n=68 because of 3 missing data points.

Note: Data are presented as n (%).

Table 5 presents means and SDs for patient and provider ratings of depression and anxiety symptoms during the pandemic. Average patient ratings of depression and anxiety did not differ substantially between visit types during the pandemic (*P*=0.418 and *P*=0.167, respectively). Patient depression and anxiety symptom ratings were significantly correlated (r=0.57, *P*<0.001). Patient self-rated anxiety and depression were not significantly correlated with the type of appointment most recently attended (*P*=0.606 and *P*=0.312, respectively). Patient self-rated anxiety and depression were also not significantly correlated with visit type preference during or after the pandemic (*P*=0.114 and *P*=0.426, respectively). The mean provider rating for depression symptoms did not increase during the pandemic, but the mean rating for anxiety symptoms did.

**Table 5.** Patient and Provider Affective Symptom Ratings

Group	Depression Symptoms	Anxiety Symptoms
Patient self-ratings		
Telehealth patients	3.92 ± 0.91	4.32 ± 0.78
In-person patients	3.73 ± 0.98	4.03 ± 0.89
All patients	3.84 ± 0.94	4.19 ± 0.83
Provider self-ratings	2.91 ± 0.54	3.91 ± 0.94

Notes: Anxiety and depression were assessed with 2 items that asked participants to rank their symptoms on a 5-point scale from “much less anxiety/depression than normal for me” to “much more anxiety/depression than normal for me.” Participants were also given a “prefer not to answer” option for these items. Data are presented as mean ± SD.

## DISCUSSION

The present study sought to investigate the impact of the COVID-19 pandemic on patient and provider experiences of and preferences for mental health appointments. While some results of our study were in line with our study hypotheses, this study had several unexpected findings. Regarding visit convenience, patients overall found both in-person and telehealth visits were convenient, inconsistent with the authors’ hypothesis. Most patients (>80% in both groups) found both visit types either “somewhat” or “very” convenient. Consistent with author hypotheses, providers rated telehealth visits as significantly more convenient than in-person visits during the pandemic, and the majority preferred telehealth visits to continue during the pandemic.

Regarding patients’ interaction with their provider, we found no difference in comfort levels or ease of interaction between telehealth and in-person visits, with both visit types rated overall “somewhat easier or more comfortable” compared with prior in-person appointments. These findings may suggest that patients did not feel that modified components of in-person visits, such as masking and screening procedures, were significantly negatively impactful on their experience with their provider. In contrast, consistent with the authors’ hypothesis, providers overall also tended to rate higher comfort and easier interaction with patients via telehealth visits during the pandemic compared with in-person visits during the pandemic.

Regarding visit preferences during and after the pandemic, patients during the pandemic reported a stronger preference for telehealth visits compared with in-person visits; however, a substantial percentage of patients also cited no preference. This preference was not sustained for patients after the pandemic, with more patients reporting a preference for in-person visits than telehealth. Overall, this finding was consistent with the authors’ hypothesis. Following the end of the pandemic, more providers indicated a preference for in-person visits than telehealth (33.3% vs 16.7%, respectively), but the majority (50%) stated no preference.

Many of these findings are consistent with a study regarding preferences for telehealth visits during the pandemic.^10^ In a study of 244 participants, Severe et al found that a majority of patients rated positive experiences with telehealth visits (telephone or video) during the pandemic, with about half of respondents reporting interest in continuing telepsychiatry visits vs in-person visits when available. Convenience and reduced risk of contracting the COVID-19 virus were cited as factors in the choice.^10^

Regarding depression and anxiety ratings, findings were largely inconsistent with the authors’ hypothesis. While patients did report higher levels of depression and anxiety during the pandemic, how patients rated these symptoms during the pandemic did not correlate with their visit preferences (telehealth or in-person) during or after the pandemic, suggesting that higher COVID-19–related depression or anxiety did not impact patients’ preferences for a particular visit type.

Limitations of the present study include the small sample size in both participant groups, especially the provider group. Another limitation is the low response rate in the patient group. These data represent a population from a single outpatient practice. As such, generalizability beyond the study area is limited. Additional studies with multisite involvement would provide larger sampling and allow for generalizability of study results. Limitations may also be present within the patient group among those without access to telehealth visits (lack of access to computer, internet) that may introduce bias. Other factors such as difference in age groups (older vs younger) and socioeconomic status may also play a role; however, these hypotheses were not explored in the present study.

## CONCLUSION

The COVID-19 pandemic prompted a significant and rapid shift toward telehealth and telepsychiatry practices and may be a catalyst for widespread and lasting adoption of telemedicine across the mental health field. However, despite this change, the present study found many patients and some providers still prefer in-person visits. This information offers insight to how we may continue to evolve clinical services offered to patients and demonstrates roles for both in-person and telepsychiatry services.

## Notes

*This article meets the Accreditation Council for Graduate Medical Education and the American Board of Medical Specialties Maintenance of Certification competencies for Patient Care and Medical Knowledge.*
